# Supplementary material for: Unassisted selective solar hydrogen peroxide production by an oxidised buckypaper-integrated perovskite photocathode
Source: Nat Commun. 2021 Nov 17;12:6644. doi: 10.1038/s41467-021-26832-5 (PMC8599672; doi:10.1038/s41467-021-26832-5)
Supplement: Supplementary file 1 — Supplementary Information [file 41467_2021_26832_MOESM1_ESM.pdf]

Supplementary Information

Unassisted selective solar hydrogen peroxide production by an oxidised buckypaper-integrated perovskite photocathode

Rashmi Mehrotra [1,2]†, Dongrak Oh[1,2]†, Ji-Wook Jang[1,2,3]*

[1] School of Energy and Chemical Engineering, Ulsan National Institute of Science and Technology (UNIST), Ulsan 44919, Republic of Korea.

[2] Department of Energy Engineering, Ulsan National Institute of Science and Technology (UNIST), Ulsan 44919, Republic of Korea

[3] Emergent Hydrogen Technology R&D Centre, Ulsan National Institute of Science and Technology (UNIST), Ulsan 44919, Republic of Korea

†These authors contributed equally to this work

*Email: jiwjang@unist.ac.kr (J.-W.J.)

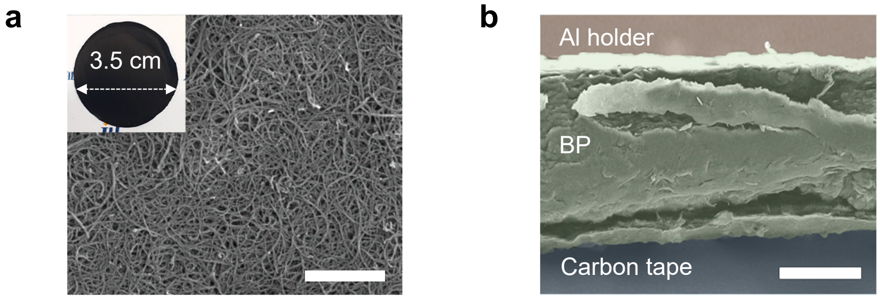

Supplementary Fig. 1 | Scanning electron micrographs of buckypaper (BP). a, Top-view scanning electron micrograph of BP. Inset, BP after being removed from the polytetrafluoroethylene membrane. Scale bar, 1 μm. **b**, Cross-section scanning electron micrograph of BP. Scale bar, 100 μm.

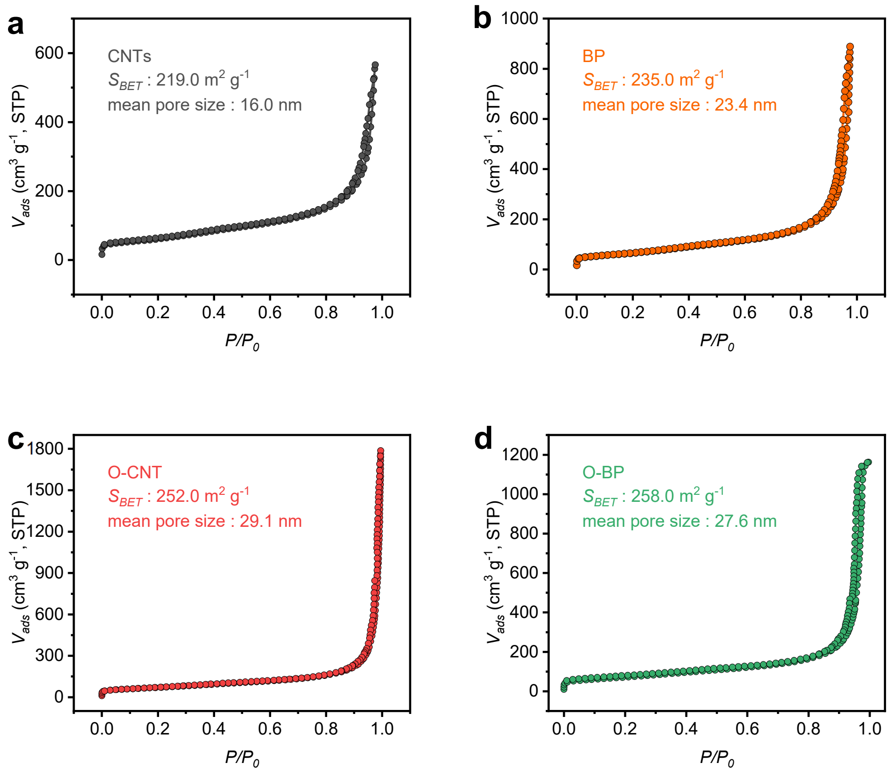

Supplementary Fig. 2 | Physisorption Analysis. a–d, Brunauer–Emmett–Teller (BET) surface areas and mean pore sizes of the carbon materials: carbon nanotubes (CNTs) (**a**), BP (**b**), oxidised CNTs (O-CNTs) (**c**), and oxidised BP (O-BP) (**d**).

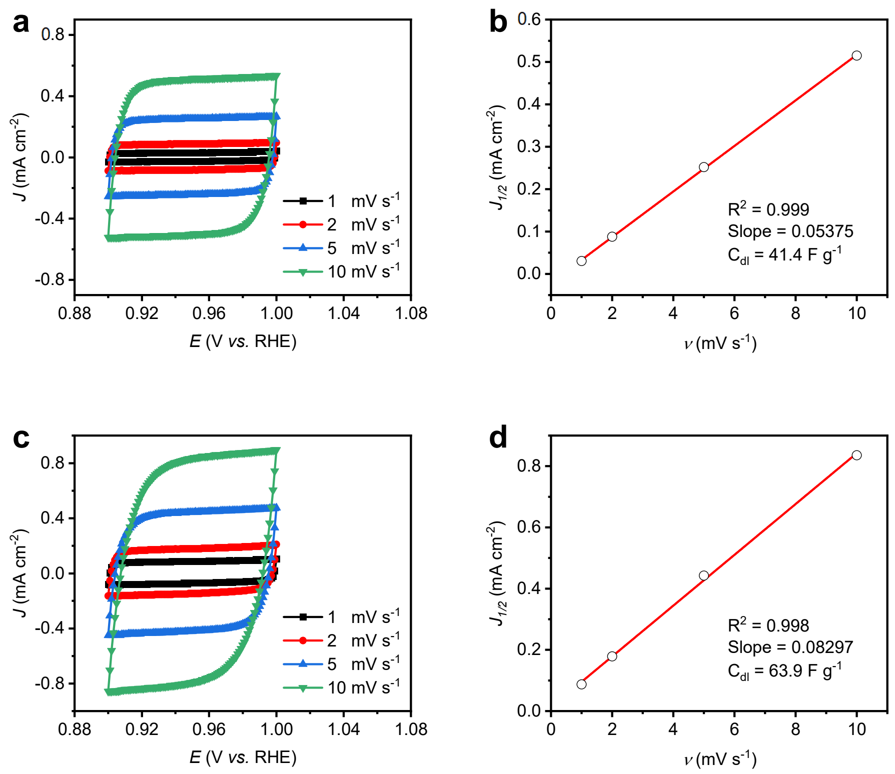

Supplementary Fig. 3 | Double-layer capacitance measurements. a–d, Electrochemical double layer capacitances (C_{dl}) of BP (**a**,**b**) and O-BP (**c**,**d**) were determined using the mid-point of J ($J_{1/2}$, at 0.95 V *vs.* RHE) within a potential window where the faradaic reaction does not occur. Typically, a series of cyclic voltammograms (CV) were obtained at various scan rates (1, 2, 5, 10 mV s^{-1}) within the potential range of 0.9–1.0 V (*vs.* RHE). It was assumed that (1) the obtained CV is rectangular-like in shape and (2) the carbon sheets have a diameter of 3.5 cm and weight of 50 mg.

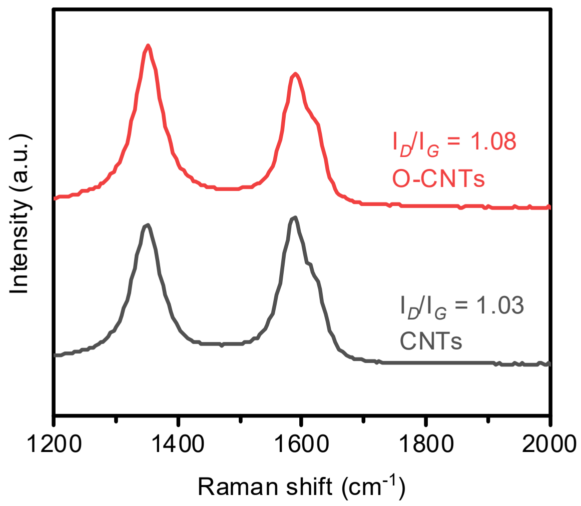

Supplementary Fig. 4 | Raman spectra of carbon materials.

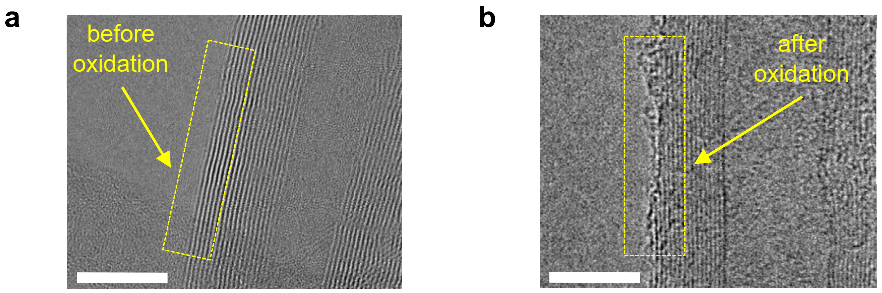

Supplementary Fig. 5 | High-resolution transmission electron micrographs. a, CNTs and **b**, O-CNTs. **a**,**b**, Scale bar, 10 nm.

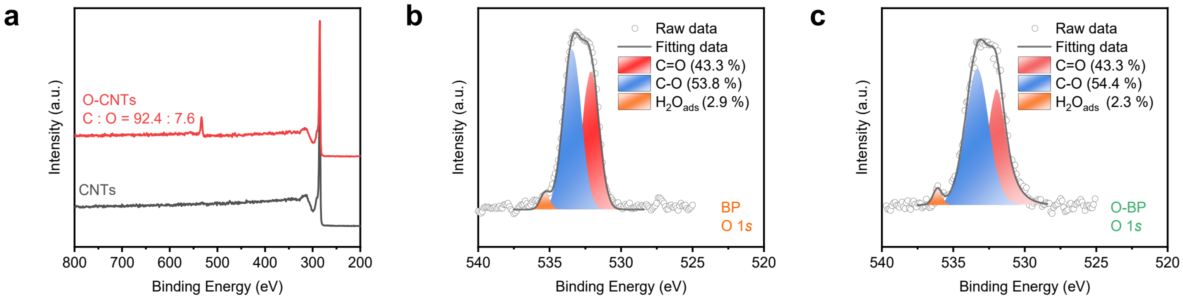

Supplementary Fig. 6 | X-ray photoelectron spectroscopy (XPS) surveys of the carbon materials. a, XPS surveys of the CNTs and O-CNTs. **b**,**c**, Deconvoluted oxygen 1s spectra of BP (**b**) and O-BP (**c**). The O 1*s* peaks of BP (O-BP) were deconvoluted into three peaks with the components centred at 532.1 (532.0) eV, 533.4 (533.4) eV, and 535.5 (536.0) eV and were assigned to C=O and C–O surface functional groups, and adsorbed water, respectively.

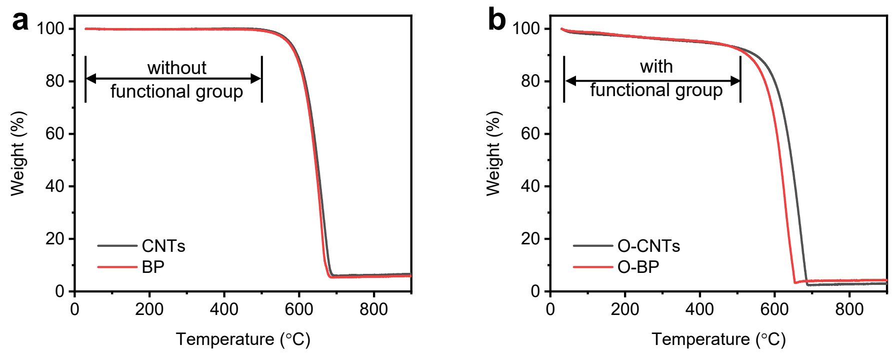

Supplementary Fig. 7 | Thermogravimetric analyses. a, CNTs with BP. **b**, O-CNTs with O-BP.

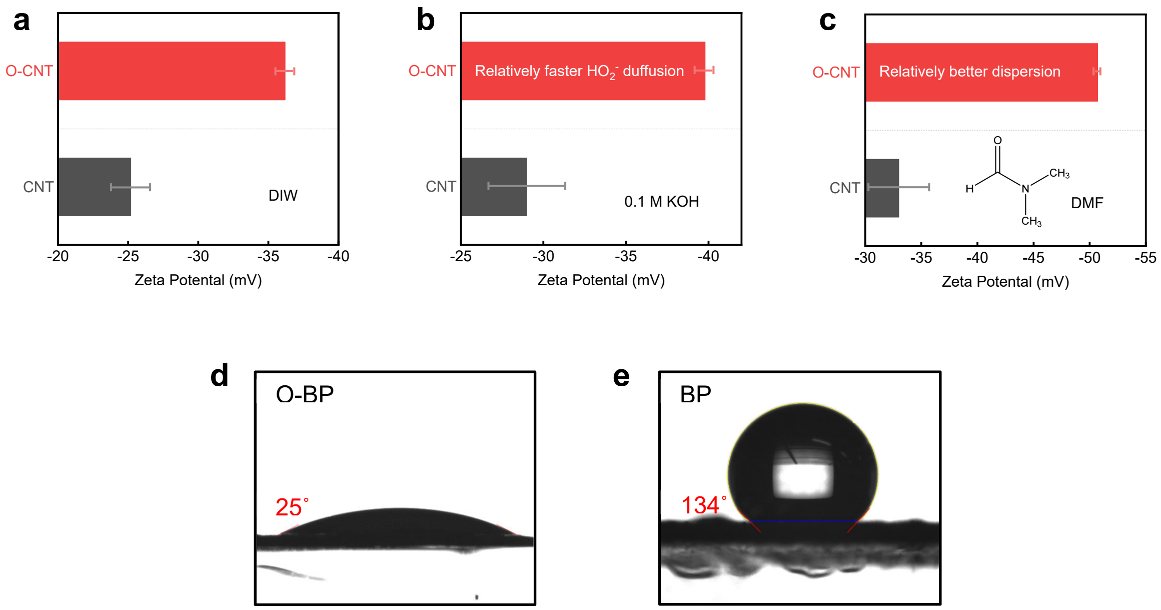

Supplementary Fig. 8 | Zeta-potentials of the carbon materials. Zeta-potentials of the CNTs and O-CNTs in **a**, deionised water, **b**, KOH, and **c**, DMF. A higher absolute zeta-potential value indicates better dispersivity. The more negatively charged O-BP woven by the O-CNTs facilitates the unhinging of the generated HO_2^- anions. **a**–**c**, The error bars indicate standard deviation. **d**,**e**, Contact angle measurements of O-BP (**d**) and BP (**e**).

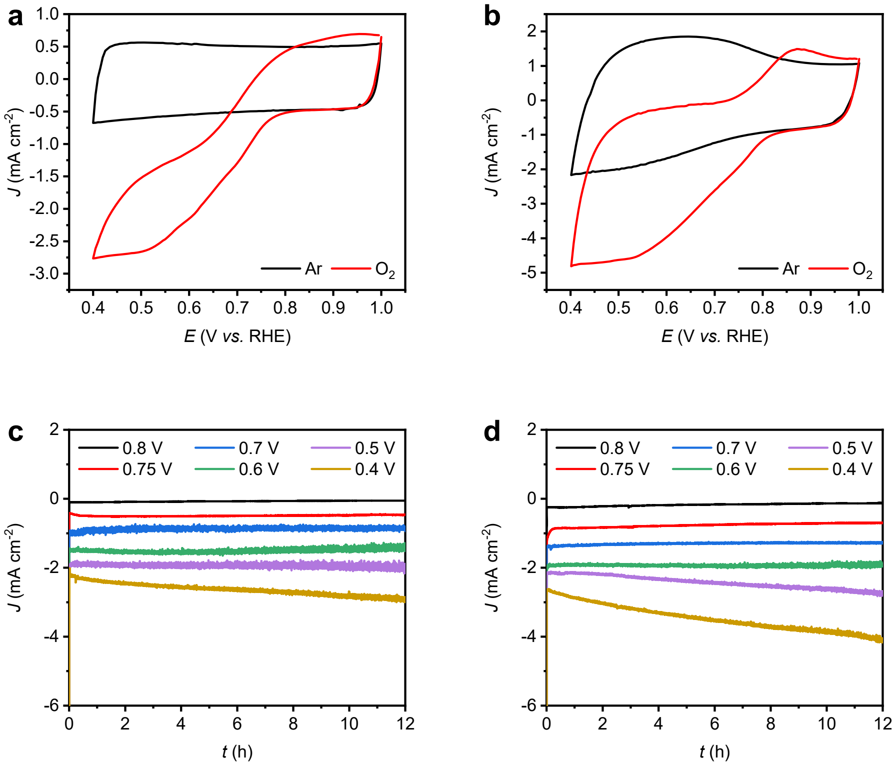

Supplementary Fig. 9 | Oxygen reduction performance of BP and O-BP. a,**b**, Cyclic voltammograms of BP (**a**) and O-BP (**b**). **c**,**d**, Chronoamperometry of BP (**c**) and O-BP (**d**) under several controlled-potential electrolyses (CPE) for 12 h. The current density at high overpotential continuously increases and the hydrogen peroxide (H_2O_2) gradually accumulates with time, potentially leading to an increase in the H_2O_2 reduction rate. **a–d**, All experiments were conducted in a 0.1 M KOH solution (~pH 13.17) under an O_2 environment and ambient conditions with vigorous stirring.

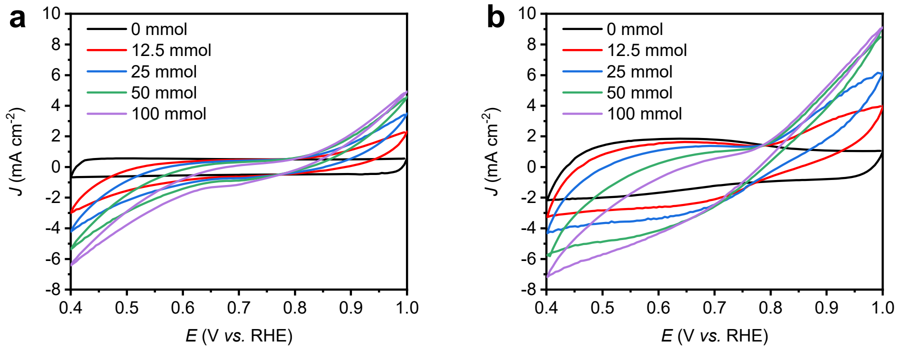

Supplementary Fig. 10 | H_2O_2 reduction performance of BP and O-BP. a,**b**, CV of BP (**a**) and O-BP (**b**). All experiments were conducted in a 0.1 M KOH solution (pH ~ 13.17) using various concentrations of H_2O_2 under an Ar atmosphere and ambient conditions with stirring.

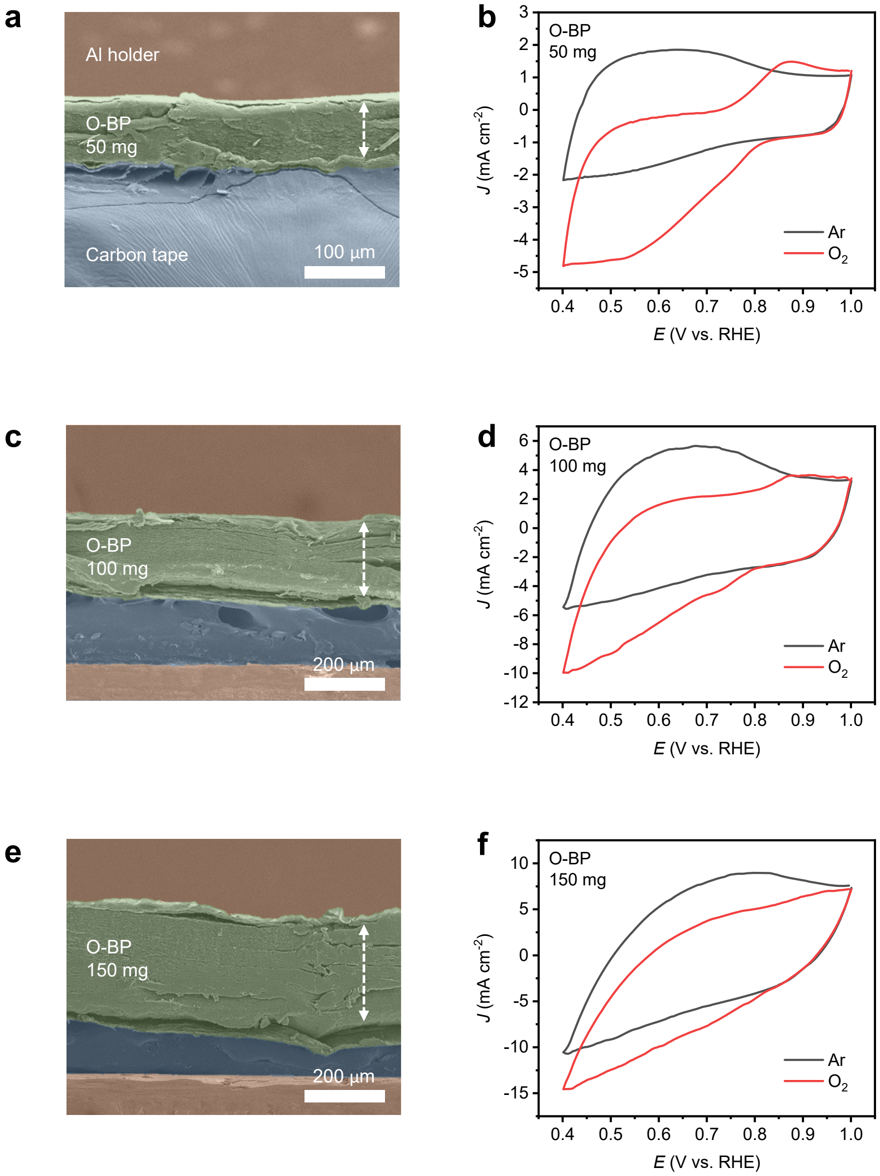

Supplementary Fig. 11 | Cross-sectional scanning electron microscopy (SEM) images of O-BP. a,**c**,**e**, Cross-sectional SEM images of O-BP in amounts of 50 mg (**a**), 100 mg (**c**), and 150 mg (**e**). **b**,**d**,**f**, Corresponding cyclic voltammograms of O-BP in amounts of 50 mg (**b**), 100 mg (**d**), and 150 mg (**f**) for determining capacitance in addition to the oxygen reduction reaction (ORR) activity.

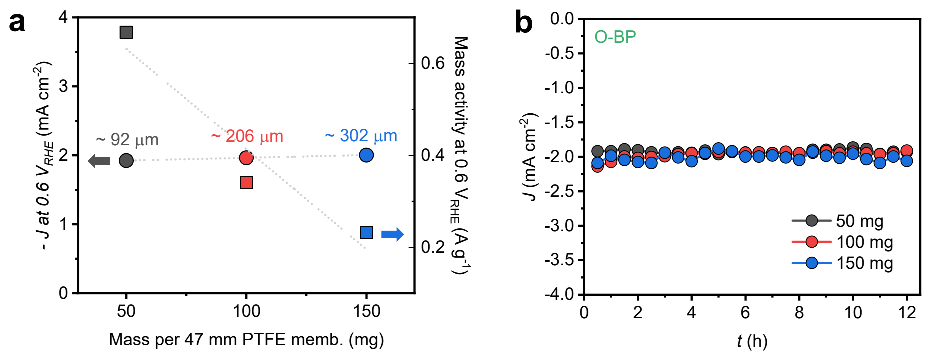

Supplementary Fig. 12 | Optimisation for O-BP thickness on ORR activity. a,**b**, Optimisation for ORR activity (**a**) and the results of 12 h of stability tests (**b**) for 50 mg (dark grey), 100 mg (red), and 150 mg (blue) of O-BP. Every experiment was conducted at + 0.6 V (*vs.* RHE) under an O_2 environment.

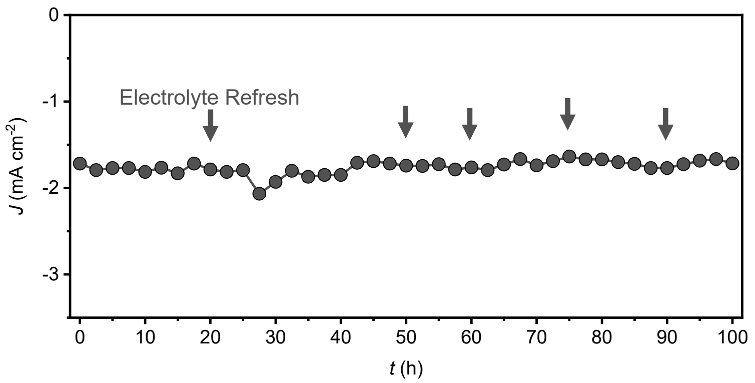

Supplementary Fig. 13 | Chronoamperometry of O-BP. Exactly 100 h of stability demonstrated by O-BP in O_2-saturated 0.1 M KOH (pH ~ 13.17) with stirring, at 0.6 V *vs.* RHE, after sufficient stabilization (~0.5 h). This experiment was conducted without a Nafion 117 membrane (Dupont) to prevent H_2O_2 accumulation.

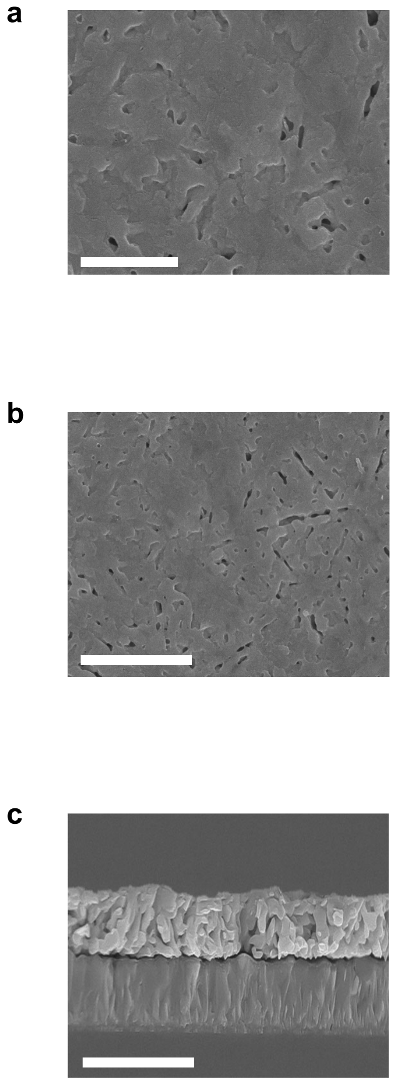

Supplementary Fig. 14 | Scanning electron micrographs of lead iodide (PbI_2). a,**b**, Top-view micrographs. **c**, Magnified cross-sectional micrograph. **a**, Scale bar, 1 µm. **b**,**c**, Scale bars, 2 µm.

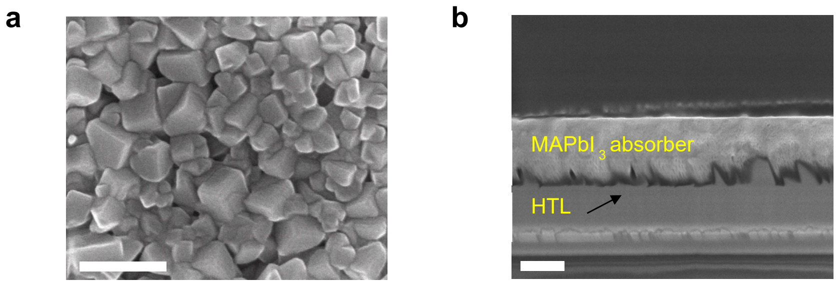

Supplementary Fig. 15 | Scanning electron micrographs of the methylammonium lead triiodide ($MAPbI_3$) perovskite (PSK) photoabsorber. a, Top-view micrograph. **b**, Cross-sectional micrograph focusing on the intercalation of methylammonium (MAI) onto the pores of PbI_2 to form $MAPbI_3$. **a**,**b**, Scale bars, 500 nm.

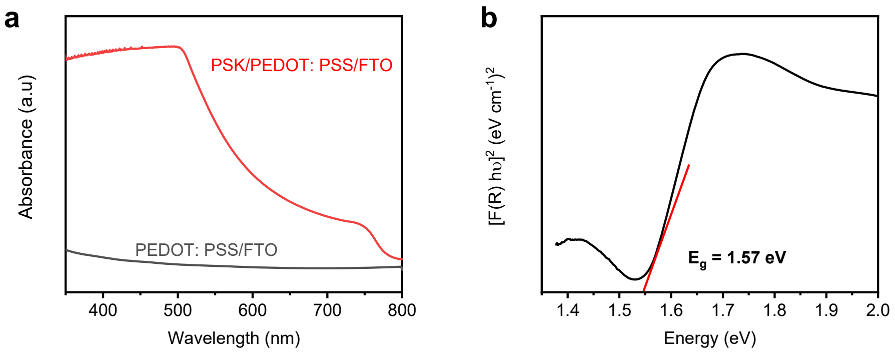

Supplementary Fig. 16 | Optical property of $MAPbI_3$ PSK photoabsorber. a, UV-vis spectra of PSK/poly(3, 4-ethylenedioxythiophene) polystyrene sulfonate (PEDOT:PSS)/fluorine-doped tin oxide (FTO) (red) and PEDOT:PSS/FTO (black), showing the absorption band at 775 nm in the formed layer. **b**, PSK optical band gap (Eg) evaluated by diffusive reflectance spectroscopy (DRS) is 1.57 eV.

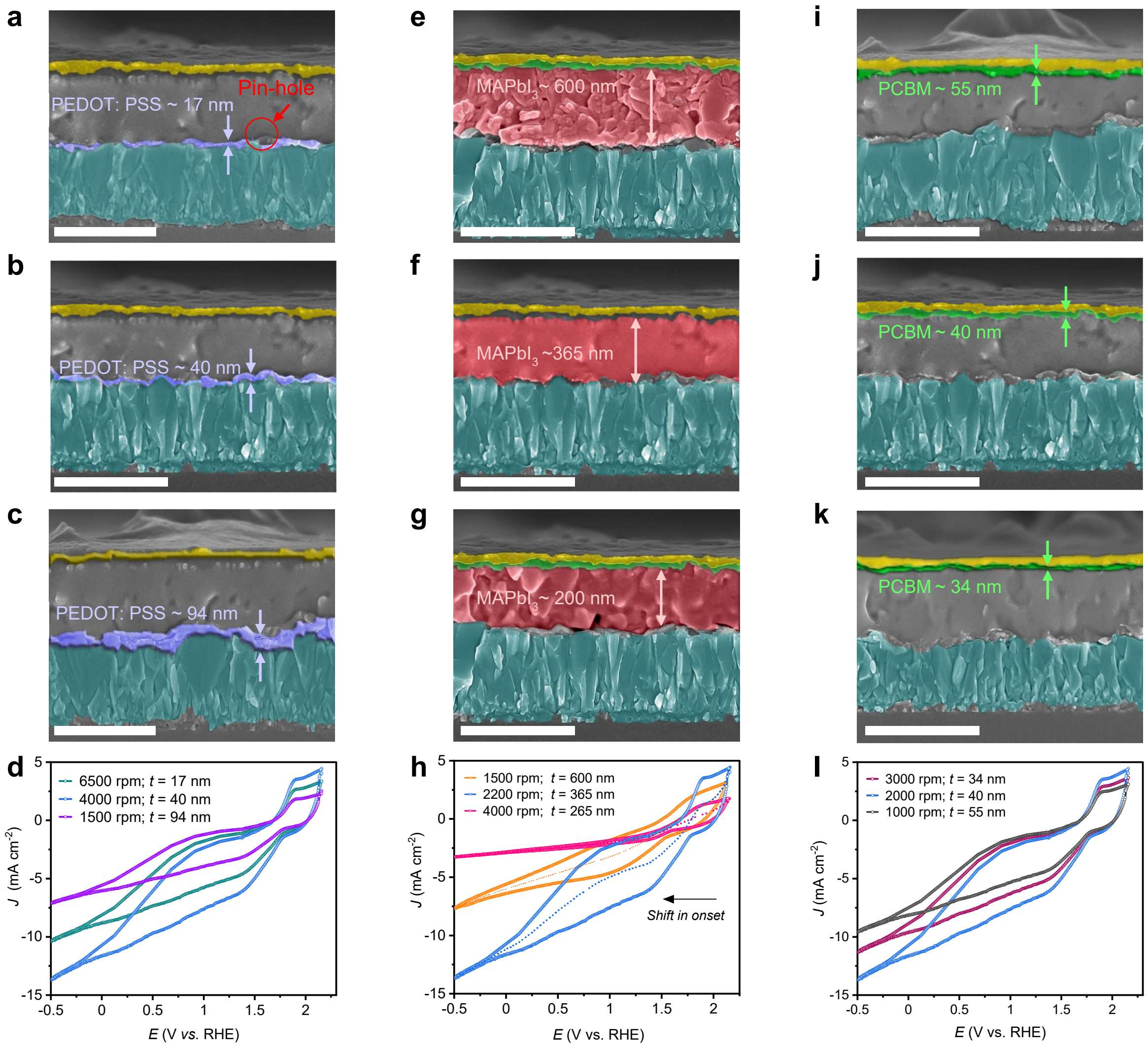

Supplementary Fig. 17 | Cross-sectional scanning electron microscopic images of $MAPbI_3$. **a–c**, Variation in the thickness of the HTL (with other layer conditions kept constant) due to a change in the spin-casting speed (rpm). Scale bars, 1 µm. **d**, The corresponding effect of the thickness on the performance of the integrated O-BP/Field's metal (FM)/PSK device. **e–g**, Variation in the thickness of photoabsorber (with other layer conditions kept constant) due to a change in the spin-casting speed (rpm); this also reduced the substrate temperature and thus changed the morphology. Scale bars, 1 µm. **h**, Corresponding effect of thickness on the

performance, which shows that optimised thickness is necessary when the precursor ink is dispensed. **i–k**, Variation in the thickness of the electron transfer layer (ETL) (with other layer conditions kept constant) due to a change in the spin-casting speed (rpm). Scale bars, 1 μm. **l**, Since the ETL was not very thick, the variation in the performance was negligible as compared to the changes observed with the variations caused by changing the thickness of the HTL and PSK photoabsorber.

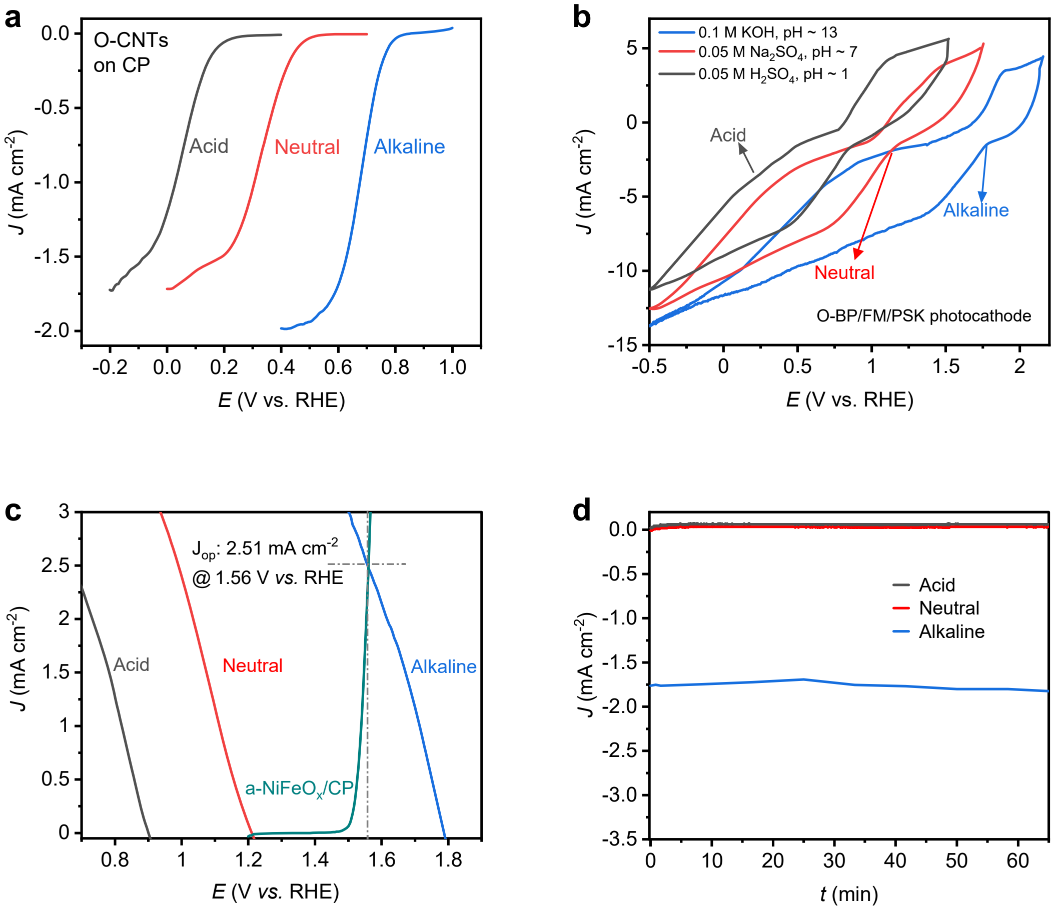

Supplementary Fig. 18 | Performance measurement of O-BP/FM/PSK photocathode device in acid, neutral, and alkaline media. a, Linear sweep voltammetry (LSV) measurements of the O-CNTs evaluated at a 5 mV s^{-1} scan rate. **b**, *J–E* response of the integrated O-BP/FM/PSK photocathode device in acidic (black colour), neutral (red colour), and alkaline (blue colour) media. Integrated O-BP/FM/PSK photocathode devices were simulated under 1-sun illumination and an air mass of 1.5 G in the presence of an O_2 supply. **c**, Operating point from the overlap of the *a*-$NiFeO_x$/carbon paper (CP) anode and average cyclic voltammogram scan of the integrated O-BP/FM/PSK photocathode in acidic (pH ~ 1), neutral (pH ~7), and alkaline (pH ~ 13) media, respectively. **d**, Chronoamperometry test of O-BP/FM/PSK at 0 V *vs.* counter electrode.

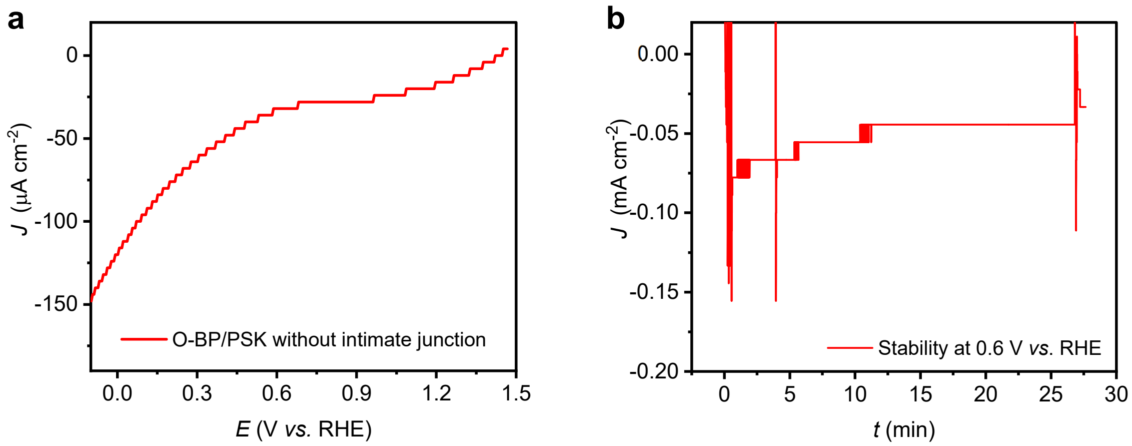

Supplementary Fig. 19 | Performance measurement of the FM/PSK photocathode. a, LSV measurements of the FM/PSK device without the integration of the O-BP as an electrocatalyst. **b**, Stability measurements at 0.6 V *vs.* RHE show an unstable photocurrent during the reaction. **a**,**b**, Experiments conducted in a 0.1 M KOH solution (pH ~13.17) under ambient conditions at a scan rate of 5 mV s^{-1} and with a continuous O_2 supply.

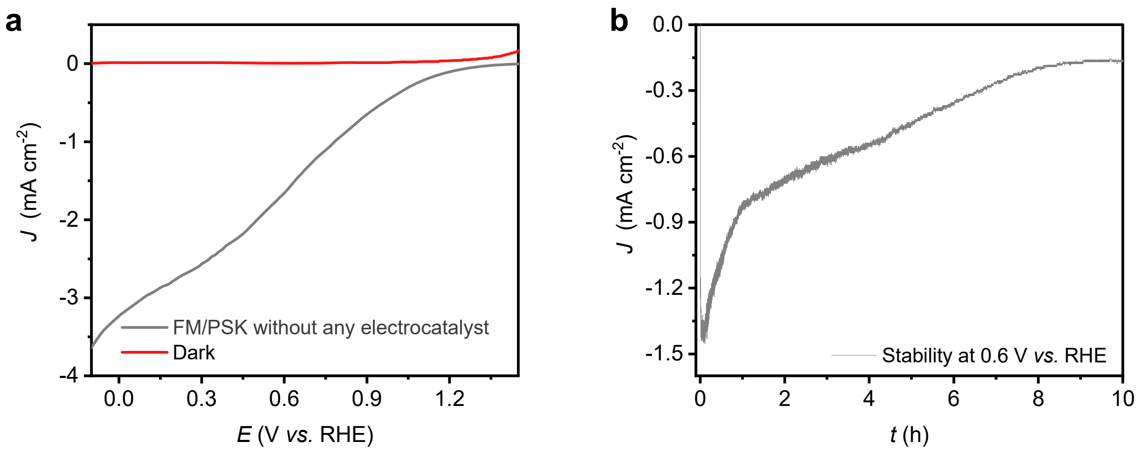

Supplementary Fig. 20 | Performance measurement of the FM/PSK photocathode. a, LSV measurements of the FM/PSK device without the integration of the O-BP as an electrocatalyst. **b**, Stability measurements at 0.6 V *vs.* RHE show an unstable photocurrent during the reaction. **a**,**b**, Experiments conducted in a 0.1 M KOH solution (pH ~13.17) under ambient conditions at a scan rate of 5 mV s^{-1} and with a continuous O_2 supply.

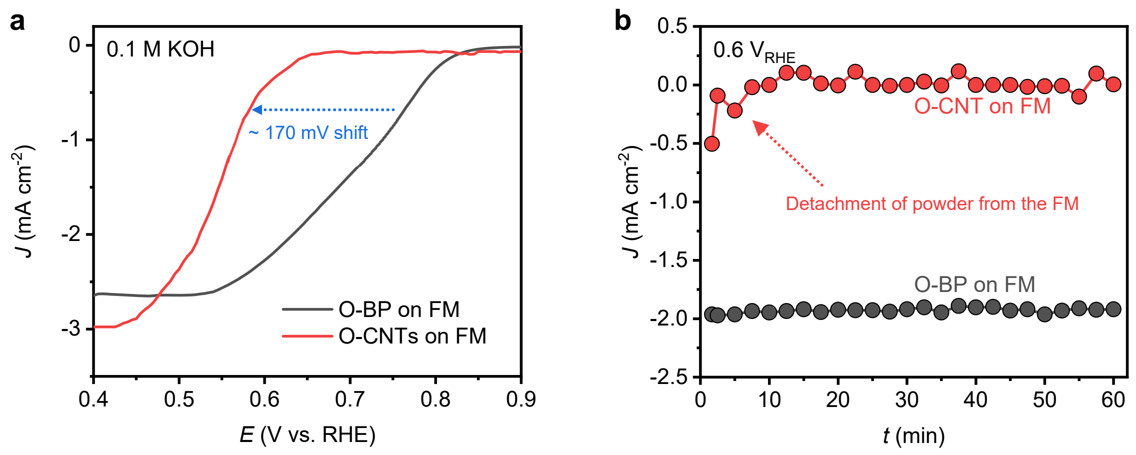

Supplementary Fig. 21 | Electrocatalytic performance measurement of powder and sheet type carbon catalysts. a,**b**, ORR activity (**a**) and chronoamperometry measurements (**b**) of sheet-type (O-BP) and powder-type (O-CNTs) carbon materials combining with FM. The weight of the powder-type carbon is the same as that of the sheet-type carbon (~ 1.3 mg in 0.25 cm^2).

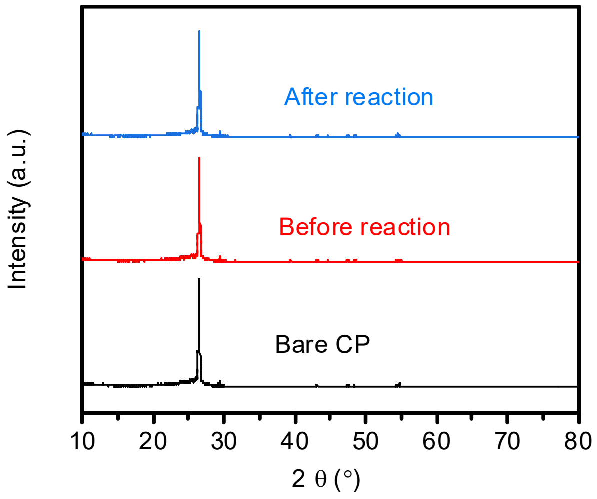

Supplementary Fig. 22 | Characterization of anode material. X-ray diffraction (XRD) patterns of the *a*-$NiFeO_x$/CP anode before (red) and after (blue) 12 h of reaction.

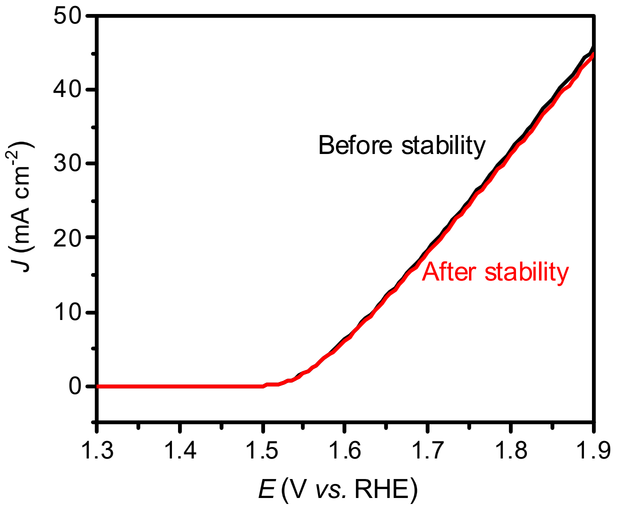

Supplementary Fig. 23 | Electrochemical performance of anode. Performance before (black) and after (red) the stability test in 0.1 M KOH solution (pH ~13.17) under ambient conditions.

.

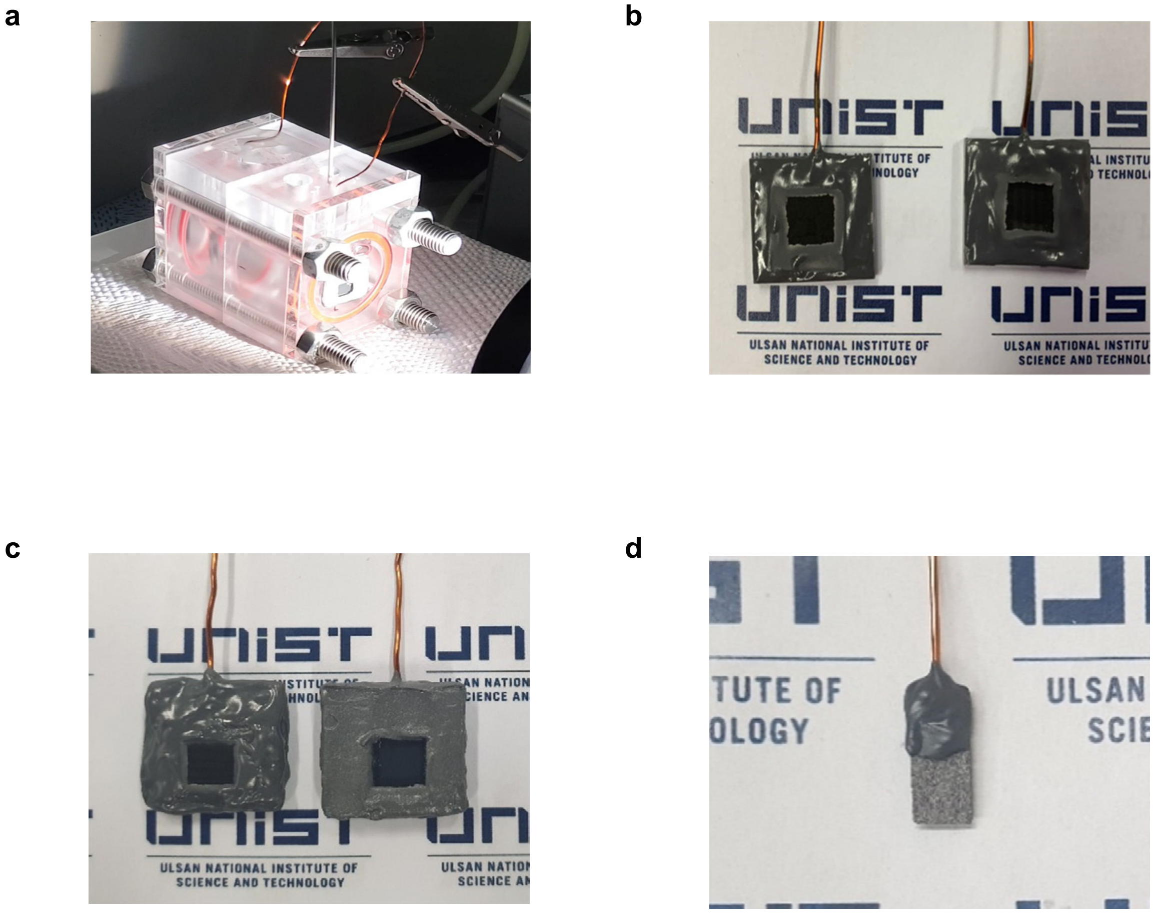

Supplementary Fig. 24 | Photographs of unassisted solar H_2O_2 system and free-standing electrodes. **a**, Side-view of the two-compartment reactor, showing the integrated photocathode device and anode directly connected by copper wires. **b**, Photograph showing the front (left) and back (right) sides of our exposed O-BP. **c**, Photograph showing the front (left) and back (right) sides of our integrated O-BP/FM/PSK photocathode. **d**, Photograph of the exposed CP coated with *a*-$NiFeO_x$ solution as the anode. Geometrical area of irradiation was afforded at ~0.25 cm^2. **a**,**b**, Each catalyst is encapsulated with epoxy resin and connected with enamelled copper wire.

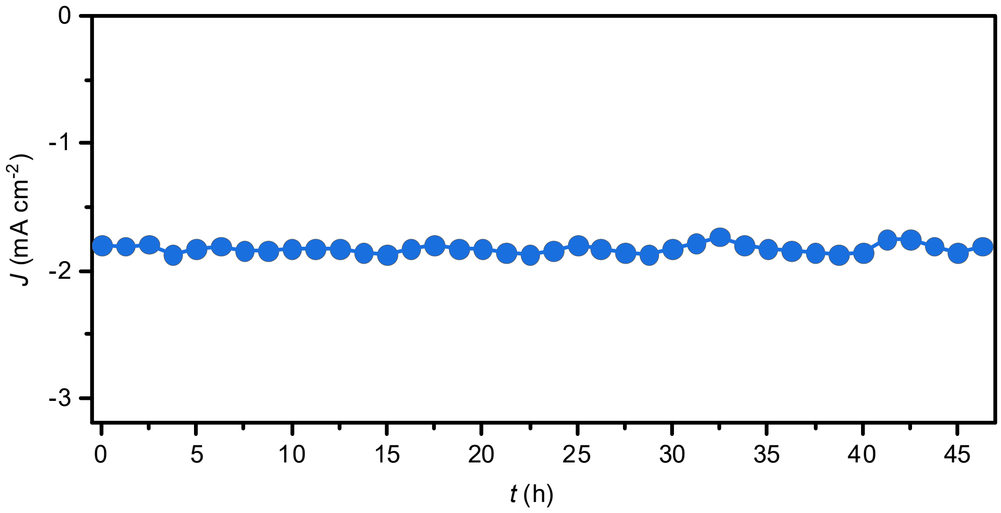

Supplementary Fig. 25 | Unassisted solar H_2O_2 generation at 0 V *vs.* counter electrode. Long-term stability tests of our integrated O-BP/FM/PSK photocathode under short-circuit conditions. Experiments were conducted in a 0.1 M KOH solution (pH ~13.17) under ambient conditions with a continuous O_2 supply.

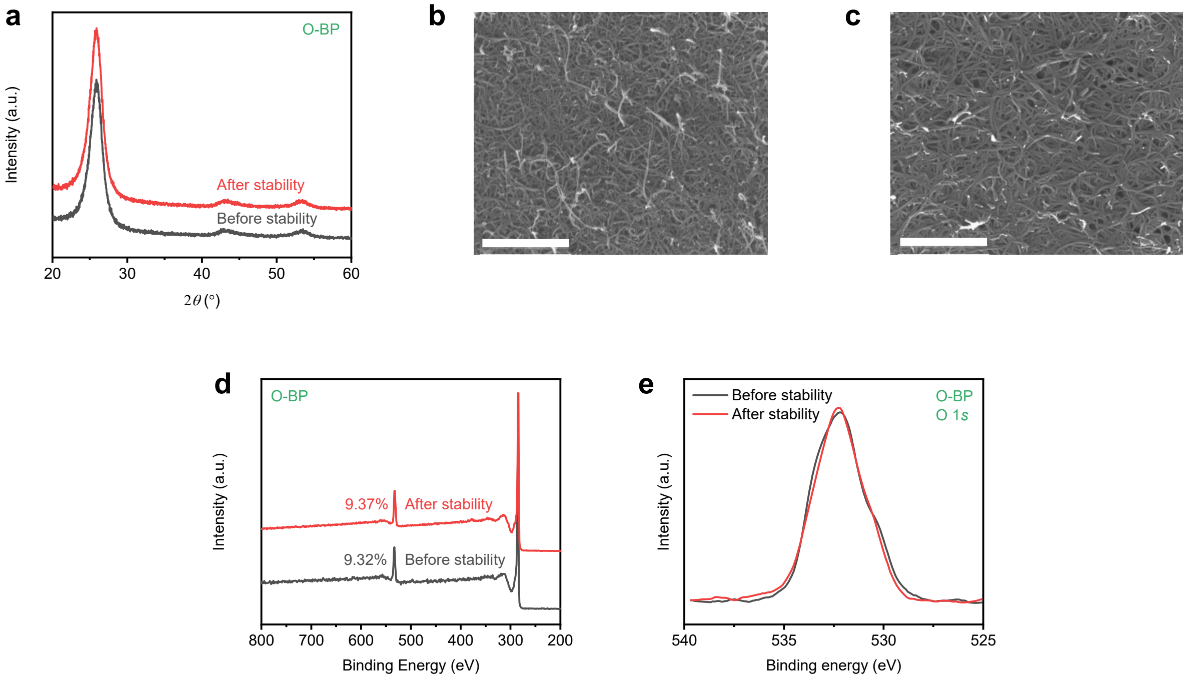

Supplementary Fig. 26 | Characterisation of the integrated O-BP/FM/PSK photocathode after measuring the long-term stability test. a, XRD patterns of the carbon materials before and after stability tests. **b**,**c**, SEM images of the O-BP before (**b**) and after (**c**) the long-term stability tests showing no accumulation of the incoming electrons at the interface rather than the rapid consumption towards the conversion into H_2O_2. **d**,**e**, XPS surveys of the carbon materials before and after stability tests. All scale bars equal 1 μm.

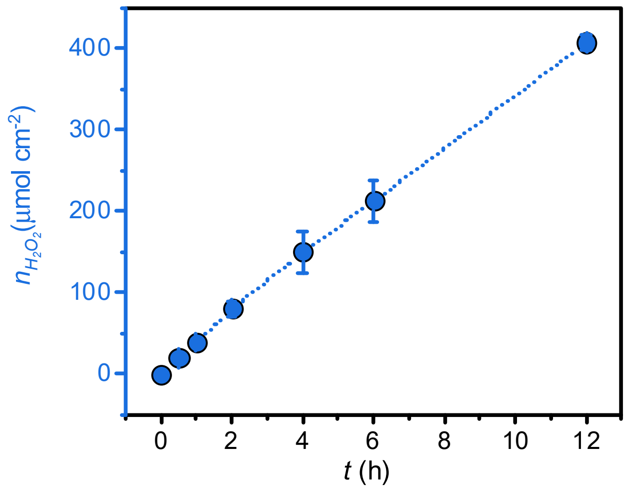

Supplementary Fig. 27 | Unassisted solar H_2O_2 generation just by connecting copper wire against the O-BP/FM/PSK *vs.* counter electrode. Amount of H_2O_2 ($\eta_{H_2O_2}$) produced by our integrated O-BP/FM/perovskite system, comprising direct copper wire connection in a two-electrode configuration, during 12 h of testing. Error bars indicate the standard deviation among the samples measured during the reaction period.

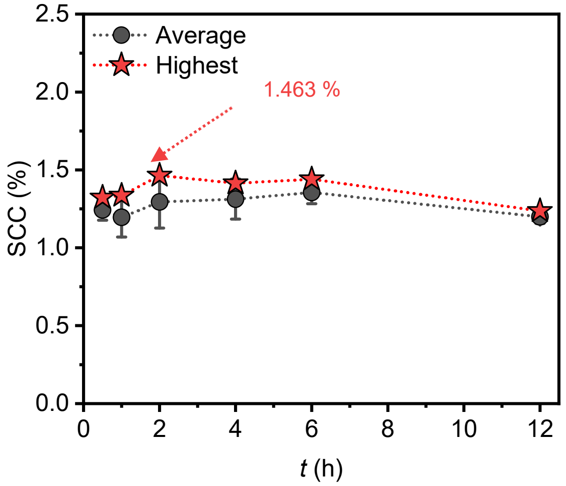

Supplementary Fig. 28 | Solar-to-chemical conversion (SCC; %) graph during 12 h of reaction time. The graph demonstrates two different values. The black circles represent the mean value along with their deviation, while the highest value attained by the best performing O-BP/FM/PSK photocathode was 1.463 % (after 2 h of reaction span) which is depicted by the red stars. Here, the error bars demonstrates the standard deviation.

a

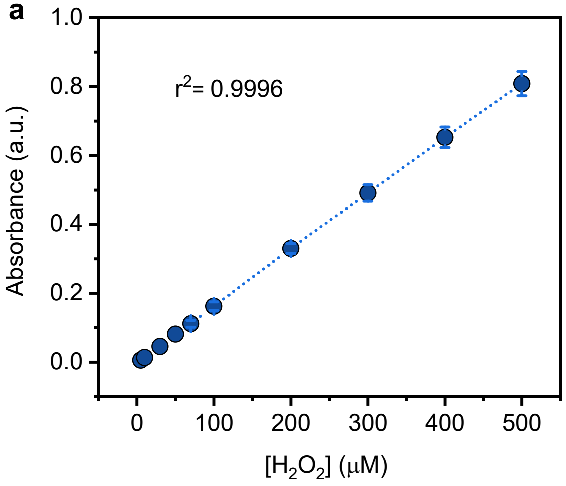

b

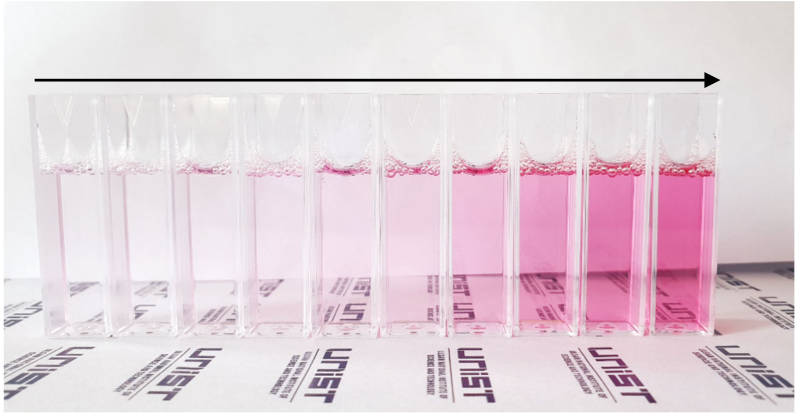

Supplementary Fig. 29 | Standard calibration curve and corresponding H_2O_2 colour. a, The standard calibration curve of H_2O_2 was determined at 551 nm by the DPD-POD method. **b**, The colour range, from left to right, in the bottom figure corresponds to the concentration change from 0 μm to 500 μm.

Supplementary Table 1 | Optimisation process conditions of all-solution processed layers including the active catalyst

Spin coating parameters		Thickness	- Jsc (mA cm^{-2})	Comments/Onset
Ramp (rpm)	Time (s)			
HTL thickness optimisation				
1500	60	94 nm	~ 5	Too thick HTL
4000	60	40 nm	~ 11.15	Optimised
6500	60	17 nm	~ 8	Non-covered area
Photo-absorber thickness optimisation				
1000	20	600 nm	~ 8.8	1.64 V *vs.* RHE
2200	20	365 nm	~ 11.15	1.77 V *vs.* RHE
4000	20	200 nm	~ 3	1.75 V *vs.* RHE
ETL thickness optimisation				
1000	30	55 nm	~ 7.8 mA cm^{-2}	No difference in the onset value
2000	30	40 nm	~ 11.15 mA cm^{-2}	
3000	30	34 nm	~ 9.2 mA cm^{-2}	
O-BP thickness optimisation at 0.6 V *vs.* RHE determining the mass activity (A g^{-1})				
Loading amount (mg)		Thickness (μm)		
50		~ 92	1.924 mA cm^{-2}	0.67 A g^{-1}
100		~ 206	1.964 mA cm^{-2}	0.34 A g^{-1}
150		~ 302	2.005 mA cm^{-2}	0.23 A g^{-1}

Supplementary Table 2 | SCC (%) and generation rates of unassisted solar H_2O_2 reported for photoelectrodes over the past five years. All the values were determined under unbiased conditions, i.e., 0 V *vs.* counter electrode applied to the two-electrode set-up

Year	Process @ 0 V vs CE	Photoelectrodes involved	Electrolyte	Generation rate (μmol cm^{-2} min^{-1})	Durability time (h)	SCC (%)	Reference
2016	ORR	WO_3‖Co^{II}(ch) on CP (generation on cathode only, 1h reaction time)	0.1 M $HClO_4$ + 0.1 M $NaClO_4$, pH 1.3	0.336	24	0.655	1
2016	ORR	WO_3/$BiVO_4$‖Au (20 min reaction time)	2 M $KHCO_3$	0.0614		0.12	2
2018	Dual	$BiVO_4$‖Carbon (400 s reaction time)	2 M $KHCO_3$, and 1 M Na_2SO_4, respectively	0.48	5	0.936	3
2019	ORR	TiO_2‖Co-N-CNT (generation on cathode only, 6 h reaction time)	0.1 M phosphate borate buffer, pH 4.5	0.035		0.069	4
2019	ORR	WO_3‖Co^{II}(ch)	$HClO_4$ (pH 1.3) + 0.1 M $NaClO_4$	0.13		0.25	5
2020	ORR	$BiVO_4$‖pTTh (utilization of both photoelectrodes)	0.1 M KOH, pH ~12.9	0.238	14	0.464	6
2020	Dual	P-Mo-$BiVO_4$‖AQ-CNT/C (5 h reaction time)	1 M $NaHCO_3$, pH ~7.8	0.16		0.312	7

2020	Dual	Mo-$BiVO_4$‖AQ-CNT/C (5 h reaction time)	1 M $NaHCO_3$, pH ~7.8	0.11			0.214		7
2021	ORR	TiO_2‖AQ-Graphite (generation on cathode only,100 h reaction time)	1 M H_2SO_4 and 1 M KOH, respectively	0.256		100	0.499		8
**2021**	**ORR**	*a*-$NiFeO_x$‖O-BP/FM/PSK (generation on photocathode only, 12 h reaction time)	**0.1 M KOH**	**Avg.**	**Best**		**Avg.**	**Best**	**This work**
				**0.637**	**0.678**	**1**	**1.19**	**1.33**	
				**0.664**	**0.751**	**2**	**1.29**	**1.463**	
				**0.695**	**0.739**	**6**	**1.35**	**1.42**	
				**0.615**	**0.634**	**12**	**1.2**	**1.24**	

Dual = water oxidation and oxygen reduction simultaneously

Reaction time = duration for which the H_2O_2 generation rate was determined

Durability time = the given system was tested at 0 V in two-electrode set-up

References

1 Mase, K., Yoneda, M., Yamada, Y. & Fukuzumi, S. Seawater usable for production and consumption of hydrogen peroxide as a solar fuel. *Nat Commun* **7**, 11470, (2016).

2 Fuku, K. *et al.* Photoelectrochemical Hydrogen Peroxide Production from Water on a WO3 /BiVO4 Photoanode and from O2 on an Au Cathode Without External Bias. *Chem Asian J* **12**, 1111-1119, (2017).

3 Shi, X. J., Zhang, Y. R., Siahrostami, S. & Zheng, X. L. Light-Driven BiVO4-C Fuel Cell with Simultaneous Production of H2O2. *Adv. Energy Mater.* **8**, (2018).

4 Ko, M. *et al.* Unassisted solar lignin valorisation using a compartmented photo-electro-biochemical cell. *Nat. Commun.* **10**, 5123, (2019).

5 Liu, J., Zou, Y., Jin, B., Zhang, K. & Park, J. H. Hydrogen Peroxide Production from Solar Water Oxidation. *ACS Energy Lett* **4**, 3018-3027, (2019).

6 Fan, W. *et al.* Efficient hydrogen peroxide synthesis by metal-free polyterthiophene via photoelectrocatalytic dioxygen reduction. *Energy Environ. Sci.* **13**, 238-245, (2020).

7 Jeon, T. H., Kim, H., Kim, H.-i. & Choi, W. Highly durable photoelectrochemical H2O2 production via dual photoanode and cathode processes under solar simulating and external bias-free conditions. *Energy Environ. Sci.* **13**, 1730-1742, (2020).

8 Jeon, T. H. *et al.* Solar photoelectrochemical synthesis of electrolyte-free H2O2 aqueous solution without needing electrical bias and H2. *Energy Environ. Sci.* **14**, 3110-3119, (2021).
